# Supplementary material for: Improvements in the sequencing and assembly of plant genomes
Source: GigaByte. 2021 Jun 10;2021:gigabyte24. doi: 10.46471/gigabyte.24 (PMC9631998; doi:10.46471/gigabyte.24)
Supplement: Figure S1 — Size distribution of reads sequenced [file gigabyte-2021-24-s001.pdf]

Figure S1: Size distribution of reads sequenced

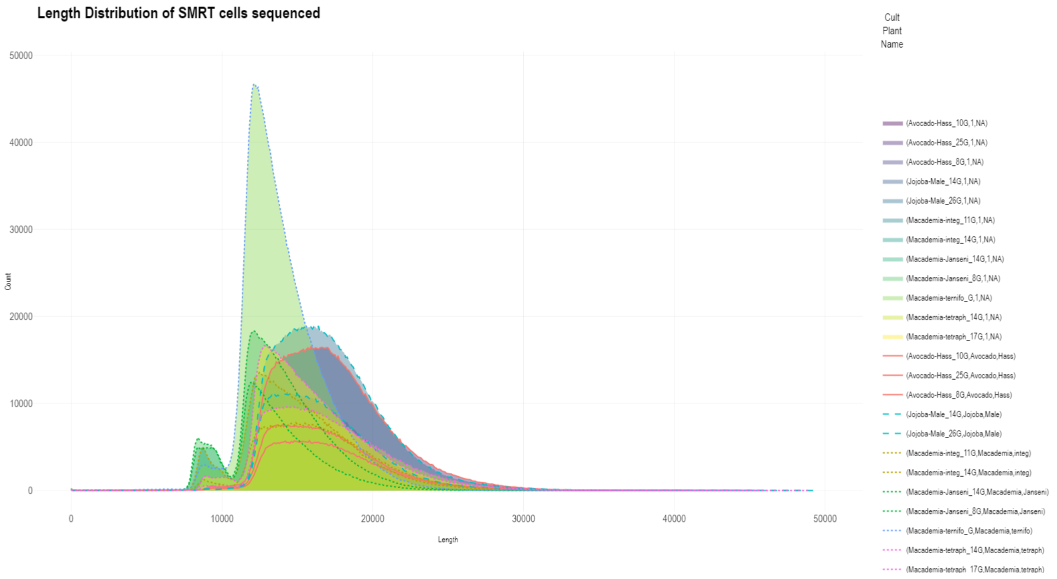

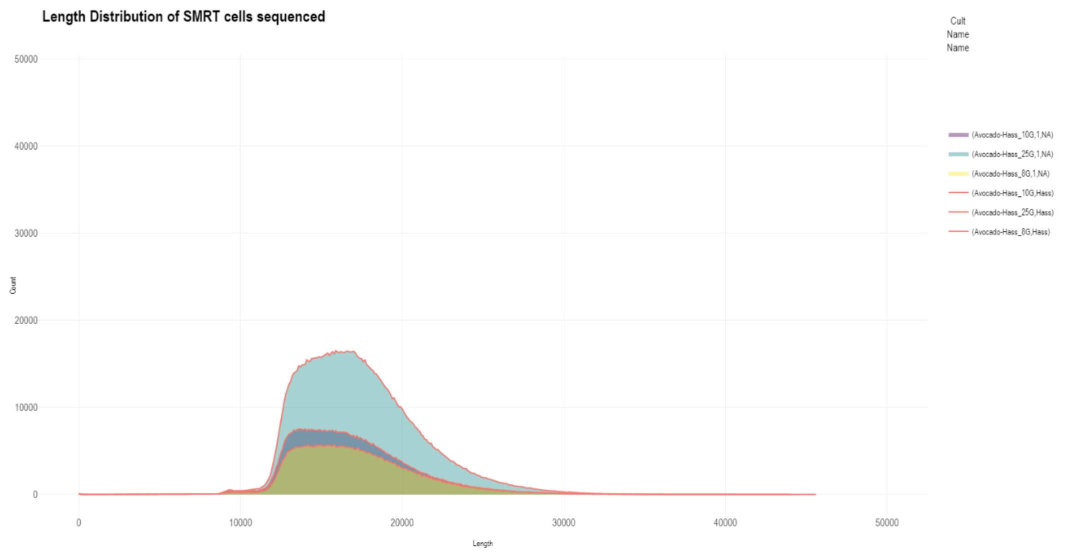
Length Distribution of SMRT cells sequenced
Cult
Name
Name
(Avocado-Hass_10G,1,NA)
(Avocado-Hass_25G,1,NA)
(Avocado-Hass_8G,1,NA)
(Avocado-Hass_10G,Hass)
(Avocado-Hass_25G,Hass)
(Avocado-Hass_8G,Hass)
Count
50000
40000
30000
20000
10000
0
0
10000
20000
30000
40000
50000
Length

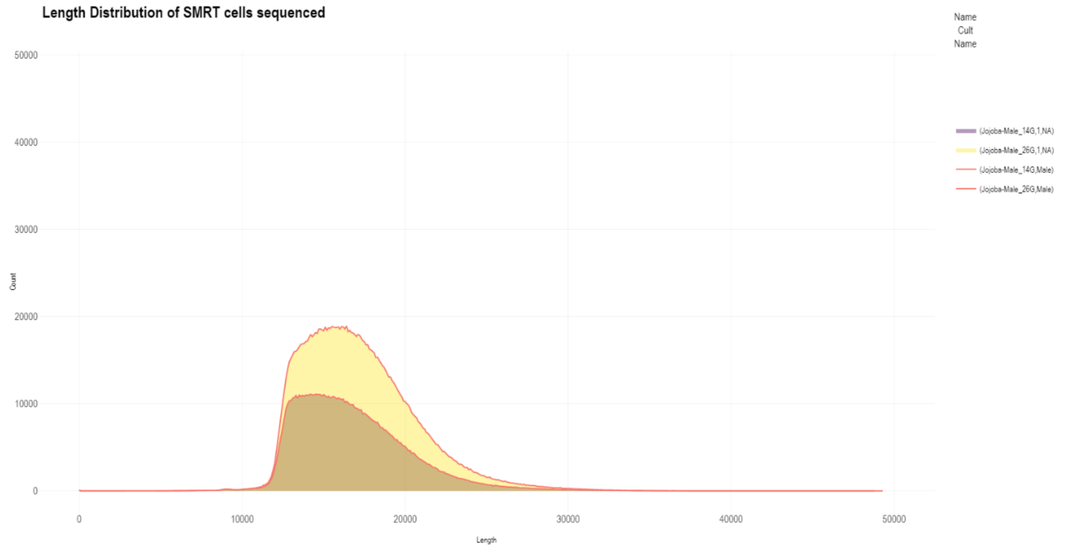
Length Distribution of SMRT cells sequenced
Name
Cult
Name
(Jojoba-Male_14G,1,NA)
(Jojoba-Male_26G,1,NA)
(Jojoba-Male_14G,Male)
(Jojoba-Male_26G,Male)
Count
50000
40000
30000
20000
10000
0
0
10000
20000
30000
40000
50000
Length

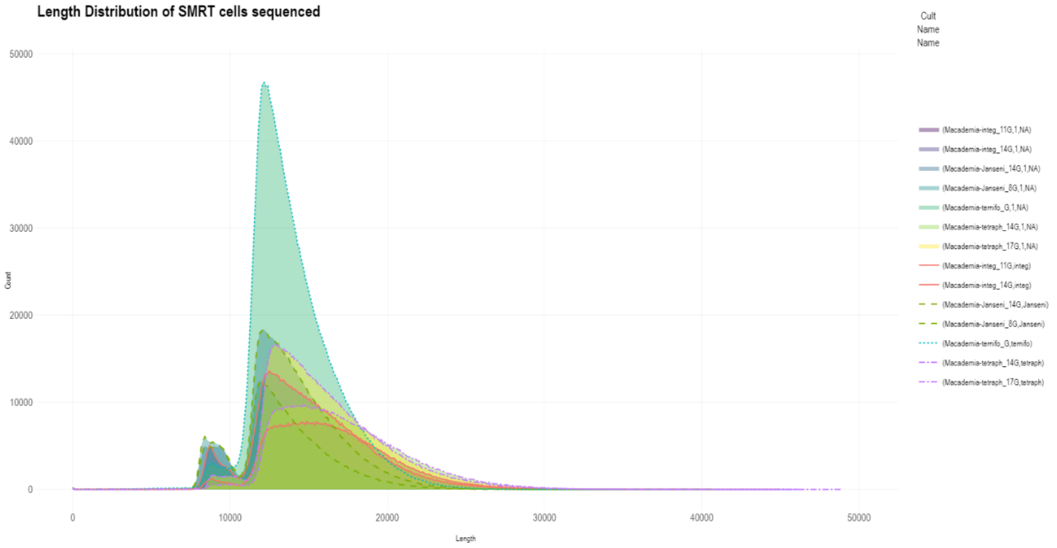
Length Distribution of SMRT cells sequenced
50000
40000
30000
20000
10000
0
Count
0
10000
20000
30000
40000
50000
Length
Cult
Name
Name
(Macademia-integ_11G,1,NA)
(Macademia-integ_14G,1,NA)
(Macademia-Janseni_14G,1,NA)
(Macademia-Janseni_8G,1,NA)
(Macademia-ternifo_G,1,NA)
(Macademia-tetraph_14G,1,NA)
(Macademia-tetraph_17G,1,NA)
(Macademia-integ_11G,integ)
(Macademia-integ_14G,integ)
(Macademia-Janseni_14G,Janseni)
(Macademia-Janseni_8G,Janseni)
(Macademia-ternifo_G,ternifo)
(Macademia-tetraph_14G,tetraph)
(Macademia-tetraph_17G,tetraph)

Supplementary Table 1 Data for associated contigs in IPA assemblies

	M. jansenii	*M. integrifolia*	*M. ternifolia*	*M. tetraphylla*	Jojoba	Avocado
Contig N50	0.45Mb	1.23Mb	0.77Mb	1.83Mb	1.69Mb	1.53Mb
Longest Contig	5.23Mb	10.22Mb	5.68Mb	14.97Mb	8.25Mb	10.0Mb
Assembly Length	527Mb	671Mb	590Mb	655Mb	738Mb	788Mb
Number of Contigs	3966	3226	3006	2103	1999	3196
